# Effect of vitamin E on sperm parameters and DNA integrity in sodium arsenite-treated rats

**Published:** 2012-05

**Authors:** Hamid Reza Momeni, Najmeh Eskandari

**Affiliations:** 1 *Department of Biology, Faculty of Sciences, Arak University, Arak, 38156-8-8349, Iran.*; 2 *Department of Biology, Faculty of Sciences, Tarbiat Moallem University, Tehran, Iran.*

**Keywords:** *Adult rat*, *DNA integrity*, *Sodium arsenite*, *Sperm parameters*, *Vitamin E*

## Abstract

**Background:** Arsenic as an environmental toxicant is able to exert malformations in male reproductive system by inducing oxidative stress. Vitamin E (Vit.E) is known as antioxidant vitamin.

**Objective:** The aim of this study was to investigate the harmful effects of sodium arsenite on sperm parameters and the antioxidant effects of Vit.E on sperm anomalies in sodium arsenite treated rats.

**Materials and Methods:** Adult male rats were divided into 4 groups: control, sodium arsenite (8 mg/kg/day), Vit.E (100 mg/kg/day) and sodium arsenite+Vit.E. Oral treatments were performed till 8 weeks. Body and left testis weight were recorded and then left caudal epididymis was cut in Ham's F10. Released spermatozoa were used to analyze number, motility, viability and abnormalities of the sperm. Sperm chromatin quality was assessed by nuclear staining using acridine orange and aniline blue.

**Results:** Body and testis weight showed no significant change in 4 groups (p>0.05). A significant decrease in the number, motility, viability and normal sperm morphology was found in sodium arsenite-treated rats compared to the control (p<0.001). Sodium arsenite had no effect on sperm DNA integrity and histon-protamine replacement (p>0.05). In sodium arsenite+Vit.E group, Vit.E could significantly compensate the harmful effects of sodium arsenite on sperm number, motility, viability and morphology compared to sodium arsenite group. In addition, sperm viability and motility was significantly increased in rats treated with Vit.E alone compared to the control and sodium arsenite+Vit.E group.

**Conclusion:** Vitamin E could compensate the adverse effects of sodium arsenite on sperm parameters in adult rats.

## Introduction

Arsenic, a sulfhydryl-reactive metalloid, is a major environmental toxicant produced by the burning of arsenic contaminated coal and glass manufacturing (1). In drinking water, inorganic arsenics are found as pentavalent (arsenate) and trivalent (arsenite) forms. In the human body, pentavalent arsenate is reduced to trivalent arsenite and it has been shown that the toxicity of arsenite compounds is higher than arsenate (2). 

Arsenic is a known human carcinogen (3) and is able to induce malformations in male reproductive system (4, 5). Beside the main source of arsenic in contaminating drinking water (1), it is used in drugs (6, 7), herbicides, insecticides and rodenticides (1, 8). Human are therefore exposed to this pollutant not only via inhalation but also via contaminated foods, water and drugs. Chronic arsenic exposure may exert serious harmful effects including cancers (9), melanosis, hyperkeratosis, lung disease, peripheral vascular disease (Blackfoot disease), gangrene (10), diabetes mellitus (11), hypertension and ischemic heart disease (10, 12). 

Arsenic can also induce male reproductive toxicity such as dose-dependent decrease in testes and accessory sex organ weights (13). It may also reduce epididymal sperm count (14), viability, and motility (15). Normal morphology (16) and the activity of antioxidant defense system (17). Alteration in the level of luteinizing hormone (LH), follicle-stimulating hormone (FSH), testosterone and also massive degeneration of the germ cells in testis tissue (14, 18, 19) is reported to be an account for arsenic toxicity. Oxidative stress and the generation of reactive oxygen species (ROS) could also be a consequence of arsenic exposure (20). ROS generation as well as the binding of arsenic to protein thiol groups can alter many protein functions (2). 

The integrity of sperm DNA is an important factor for the success of fertilization as well as normal development of the embryo, fetus and child (21). Several line of studies have shown the effect of environmental contaminates such as arsenic on DNA damages through inducing oxidative stress and the generation of ROS (3, 22, 23). 

It is now documented that vitamin E (Vit.E), as a potent antioxidant (24), protect the organism against oxidative stress via the inhibition of propagation of ROS reactions (24). In reproductive system, the antioxidant's role of this vitamin has also been reported to reduce testicular oxidative stress (25, 26). Previous studies have reported the adverse effects of sodium arsenite on adult male reproductive tract. To our knowledge, however, no study has examined the effect of Vit.E on sodium arsenite mediated toxicity in epididymal sperm of adult rat. The present study was therefore performed to examine the effect of Vit.E on epididymal sperm parameters, sperm DNA integrity and sperm histon-protamine replacement in sodium arsenite-treated adult rats.

## Materials and methods

**Animals and treatments**

This experimental study was performed on adult albino male Wistar rats (250±20 gr). The animals were purchased from Pasture's Institute, Iran. The animals were housed in plastic cages at 12-h light/dark cycle, 24±2^o^C and fed with standard commercial laboratory chew and water. 

Adult rats were divided into four groups (n=6 for each group): control which received distilled water, sodium arsenite (8 mg/kg/day, Merck, Germany), Vit.E (100 mg/kg/day, Sigma, USA) and sodium arsenite +Vit.E. The reagents were orally given to the rats by gavage for 8 weeks “as the duration of spermatogenesis in wistar rats is 52 days” (27). 

At the end of the treatments, the animals were weighed, anesthetized by the injection of pentobarbital (60 mg/kg) and sacrificed. Left testis and cauda epididymis of the animals were dissected. The testis was cleared from fat tissue and its weight was recorded.

**Sperm count**

The dissected epididymis of each animal was transferred into 10 ml Ham's F10 medium and cut to small slices, in order to swim out the sperm into the medium. After 10 min of diffusion, 1 ml of the solution was diluted with 9 ml formaldehyde fixative. The diluted solution was transferred into each chamber of Neubauer hemocytometer and sperm heads was manually counted under a microscope. Sperm count was performed according to WHO guidelines (28) and data were expressed as the number of sperm per ml.

**Sperm motility**

Assessment of sperm motility was done according to WHO protocol (28). In brief, 10 μl of the sperm suspension was placed on semen analysis chamber. A minimum of five microscopic fields were assessed to evaluate sperm motility on at least 200 sperm for each animal. The percentage of sperm motility was analyzed for following motion patterns: Progressively motile sperm (PMS), nonprogressively motile sperm (NPMS) and nonmotile sperm (NMS).

**Sperm viability**

Eosin-nigrosin staining was used to asses sperm viability according to WHO protocol (28) Briefly, eosin (1%, Merck, Germany) and nigrosin (10%, Merck, Germany) was prepared in distilled water. One volume of sperm suspension was mixed with two volume of 1% eosin. After 30 second, an equal volume of nigrosin was added to this mixture. Thin smears were then prepared and observed under a light microscope at 1000X magnification. Viable sperm remained colorless while nonviable sperm stained red.

**Sperm morphology**

The eosin-nigrosin stained slides were used to evaluate sperm morphology. One hundred sperm were observed to detect sperm abnormalities in each sample.

**Sperm chromatin quality**

Thin smears were prepared from the sperm solution and allowed to air-dry. To test sperm DNA integrity, the smears were stained with acridine orange (AO). AO staining was performed according to a protocol described by Tejada and co-workers (29). In brief, the smears were fixed for 14 hrs in methanol/acetic acid (3:1) at 4^o^C and stained with AO solution (0.19% in phosphate citrate buffer, pH=2.5) for 10 min. The slides were gently washed by distilled water for 5 minutes and air dried. The stained smears were then observed under fluorescence microscope at 1000X magnification. Three types of staining patterns were considered in sperm head; green spermatozoa (double-stranded DNA), yellow and red spermatozoa (single-stranded DNA). At least 100 spermatozoa per slide were count to evaluate the percentage of double-stranded DNA in the spermatozoa. 

To test sperm histon-protamine replacement, the smears were stained with aniline blue (AB). AB staining was carried out based on the method described by Wongand co-workers (30). In brief, the sperm smears were fixed in 4% formalin solution for 5 min, rinsed in distilled water, and stained in 5% AB in 4% acetic acid (pH 3.5) solution for 5 min. The slides were washed in distilled water, stained in 0.5% eosin for 1 min and allowed to air-dry. The slides were then examined at 1000X magnification in a light microscope. Immature sperm characterized by nuclear histone proteins stained dark blue, whereas mature sperm with protamine stained red-pink. At least 100 spermatozoa per slide were count to analyze the percentage of red-pink spermatozoa.

**Statistical analysis**

Results are expressed as mean±SD for six animals per group. One-way analysis of variance (ANOVA) was used to assess the statistical significance of the data. p<0.05 was considered significant.

## Results

**Body and testis weight**

For each animal, body and testis weight were recorded at the end of the treatments. There was no significant difference in body and testis weight in the four groups (Table I).

**Sperm count**

Results showed a highly significant decrease (p<0.001) in epididymal sperm number in sodium arsenite group compared to the control (Table II). Sodium arsenite+Vit.E group showed a highly significant increase (p<0.001) in sperm number compared to sodium arsenite group (Table II).

**Sperm motility**

The treatment of animals with sodium arsenite significantly (p<0.001) decreased the percentage of PMS and increased (p<0.001) the percentage of NPMS and NMS compared to the control (Table II). 

Rats treated with sodium arsenite+Vit.E showed a significant increase (p<0.001) in the percentage of PMS and decrease (p<0.001) in the percentage of NPMS as well as NMS as compared with sodium arsenite group (Table II). Animals treated with Vit.E alone also revealed a significant increase (p<0.001) in the percentage of PMS and decrease in the percentage of NPMS and NMS when compared with the control (Table II).

**Sperm viability**

There was a significant decrease (p<0.001) between the percentage of viable sperm in sodium arsenite group compared to the control. A significant increase (p<0.05) was observed in sperm viability in rats exposed to sodium arsenite+ VE compared to sodium arsenite group (Table II). Rats treated with Vit.E alone also showed a significant increase (p<0.05) in sperm viability when compared with the control (Table II).

**Sperm morphological anomalies**

**Figure 1:**
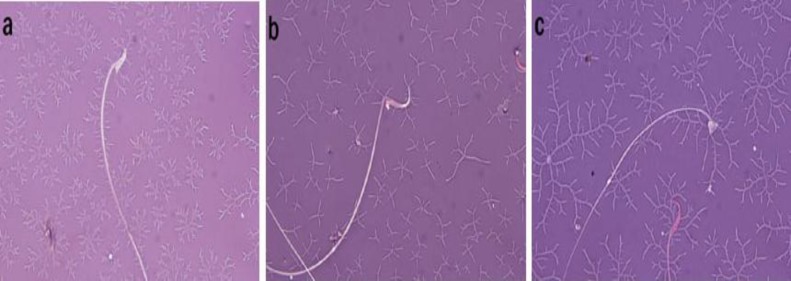
Sperm abnormalities in sodium arsenite-treated rats. a) Banana head (flattened head or reduced hook). b) Bent neck. c) Pin head. Eosin-nigrosin staining. Magnification: 1000X

Rats treated with sodium arsenite showed a significant increase (p<0.001) in abnormal sperm. In sodium arsenite+Vit.E group, Vit.E could significantly (p<0.05) reverse sperm morphological anomalies as compared with sodium arsenite group (Table II). Some sperm morphological anomalies such as banana head (Flattened head or reduced hook), pin head and bent neck induced in sodium arsenite animals are shown in Figure 1.

**Sperm chromatin quality**

**Figure 2:**
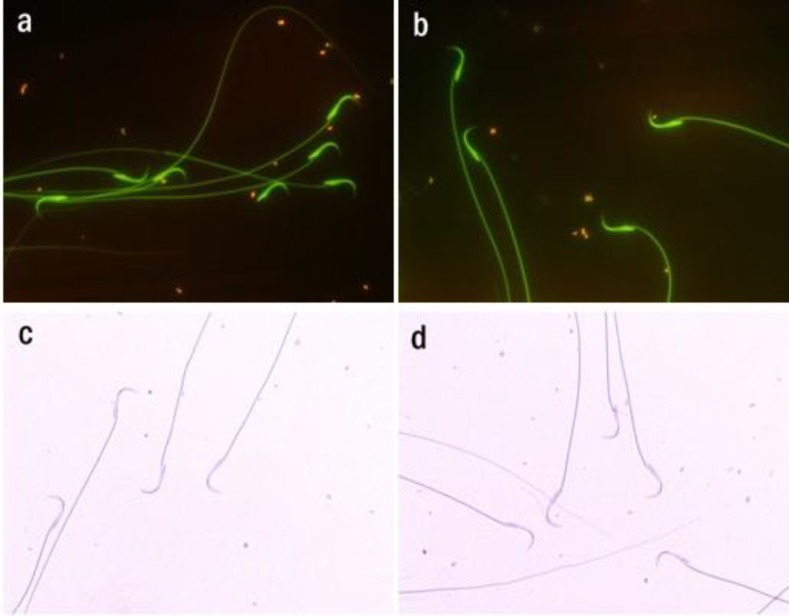
Rat spermatozoa stained with acridine orange (AO) and aniline blue (AB). AO staining: a) Control group. b) Sodium arsenite group (8 mg/kg/day). AB staining: c) Control group. d) Sodium arsenite group (8 mg/kg/day). Magnification: 1000X.

Spermatozoa stained with AO showed that sodium arsenite had no significant effect on the sperm DNA integrity compared to the control (Figure 2a-b and table III). In addition, AB staining revealed no significant effect on the histon-protamine replacement during the sperm maturation process in sodium arsenite-exposed rats compared to the control (Figure 2c-d and table III).

**Table I:** Body and testis weight

	**Control**	**Vitamin E**	**Sodium arsenite**	**Sodium arsenite+vitamin E**
Body Weight (g)	276.50±20.14	286.5±6.72	270.83±15.24	271.33±22.54
Testis Weight (g)	1.38±0.26	1.41±0.24	1.23±0.14	1.34±0.28

Mean±SD, n=6.

**Table II:** Epididymal sperm number, sperm motility, sperm viability and sperm morphological anomalies

	**Control**	**Vitamin E**	**Sodium arsenite**	**Sodium arsenite+vitamin E**
Sperm number (10^6^)	15.86±0.84	16.94±1.23	11.46±1.52*^a^	14.55±0.23*^b^
PMS%	72.24±1.7	80.89±1.31*^a^	49.69±1.55*^a^	61.22±0.85*^b^
NPMS%	16.48±1.88	11.92±0.82*^a^	28.99±1.08*^a^	24.22±1.15*^b^
NMS%	11.29±1.33	7.18±0.58*^a^	21.32±0.9*^a^	14.56±1.21*^b^
Sperm viability%	75.25±2.66	79.14±0.61**^a^	62.62±1.78*^a^	66.08±2.66**^b^
Sperm morphological anomalies%	1.12±0.23	1.07±0.39	2.66±0.75*^a^	1.84±0.4**^b^

PMS: Progressively motile sperm, NPMS: Non-progressively motile sperm, NMS: Non-motile sperm.

a : compared to control.

b : compared to sodium arsenite. Mean±SD, n=6.

* p <0.001,

** p <0.05

**Table III:** DNA integrity (acridine orange staining) and histon-protamine replacement (aniline blue staining) in rat’s epididymal sperm.

	**Control**	**Vitamin E**	**Sodium arsenite**	**Sodium arsenite+vitamin E**
DNA integrity	99.99±0.01	99.99±0.01	99.98±0.01	99.98±0.01
Histon-protamine replacement	98.34±0.08	98.37±0.05	98.28±0.09	98.3±0.07

Mean±SD, n=6

## Discussion

This study examined the adverse effect of sodium arsenite on epididymal sperm parameters in adult rats. In addition, Vit.E showed to reverse the toxic effect of sodium arsenite on these parameters.

In present study no significant difference was observed in the body and testis weight in sodium arsenite-exposed rats. Our results were in agreement with previous findings regarding the effect of sodium arsenite on the body (19) and testis (31) weight. However, certain studies have reported a reduction in the body and testis weight (4, 13) in arsenic treated animals. These different results might be due to the duration of treatment or administrated dosages (32).

In agreement with pervious study (15), our results also showed a significant decrease in the total sperm number in rats treated with sodium arsenite. One possibility for this effect might be due to a decrease in hormones such as FSH, LH or testosterone which intern reduce sperm count (14). On the other hand, it has been reported that sodium arsenite by inducing oxidative stress exerts harmful effects on organs such as testis (17). We therefore hypothesized that the toxic effect of sodium arsenite on the reduction of sperm number could be as a result of sodium arsenite-induced stress oxidative. If our hypothesis was true, Vit.E, a well-known antioxidant (24, 33), should have reversed hazardous effect of sodium arsenite on sperm number. Interestingly, we showed that in sodium arsenite+Vit.E group, Vit.E significantly ameliorated sodium arsenite-mediated decrease in sperm number. In spite of the decreased sperm number, the testis weight remained unchanged. This result suggests that the reduction in sperm number might not be as a result of testicular damages induced by sodium arsenite. Instead, other factors e.g. oxidative stress affected produced spermatozoa, leading to decreased sperm number.

Chromatin and flagellum in mammalian spermatozoa contain large amount of thiol rich protamine and sulfhydryl groups respectively which involve in the maintenance of sperm stability and motility (16). The decrease in sperm motility observed in the present study might be ascribed to the binding of arsenic to sulfhydryl or thiol groups on sperm proteins or the inhibition of enzymes involved in sperm motility (2, 16). Arsenic can also induce free radical such as ROS which exerts the peroxidation of polyunsaturated fatty acid in the sperm (5, 17). It may consequently lead to the destruction of sperm mitochondria, resulting in sperm ATP depletion (5) and reduced sperm motility and viability. It is therefore likely to assume that reduced sperm motility and viability induced by sodium arsenite has been due to the ability of this toxicant in the induction of oxidative stress. To support this idea, we showed that Vit.E, as a potent antioxidant, significantly reversed the viability and motility pattern of sperm in sodium arsenite+Vit.E group compared to sodium arsenite group. 

An interesting finding in sperm viability and motility assay was that Vit.E alone increased these parameters compared to the control. This effective result of Vit.E might also be due to its antioxidant role. This vitamin plays an important protective role for preventing the production of lipid peroxides by scavenging free radicals which are toxic for biological membranes (24). Therefore, it could be speculated that this vitamin by improving the activity of sperm defense antioxidant system including superoxide dismutase, glutathione peroxidase and catalase exerted its role in increasing sperm viability and motility. 

Our results showed a significant increase in sperm morphological anomalies in rats treated with sodium arsenite. It is documented that ROS generation can induce abnormal sperm morphology (34). It is therefore likely that ROS produced by arsenic has been responsible for sperm morphological anomalies in sodium arsenite-treated rats. The compensation of this parameter by the administration of Vit.E in sodium arsenite+Vit.E group might be attributed to the antioxidant properties of this vitamin and therefore support oxidative stress hypothesis in sodium arsenite-mediated sperm morphological anomalies.

AO and AB staining are methods for determining sperm DNA integrity (double strand DNA versus single strand DNA) (29) and histone-protamine replacement (35) respectively. Although observer subjectivity, heterogeneous slide staining, long time of fixation (in the case of AO staining) and microscopic study might be the limitations of these assesses, the methods are simple, inexpensive and still useful tools for assessing sperm chromatin structure in wide variety of basic and clinical studies (36-38). Using AO and AB staining, rats exposed with sodium arsenite displayed no significant difference in sperm DNA integrity and histon-protamine replacement. No study has examined the effect of sodium arsenite**on these parameters** in epididymal sperm of adult rat. It is likely that the effect of sodium arsenite on these parameters has been dose and duration-dependent.

Arsenic is a potent endocrine disruptor (39). Therefore, another possibility for the alteration of sperm parameters in this study might be related to this property of arsenic. Hormonal measurement in arsenic-treated rats is suggested to provide insights toward this possible mechanism.

## Conclusion

Our results indicate that sodium arsenite has a negative influence on sperm number, sperm motility, sperm viability and normal sperm morphology but not on DNA integrity and histon-protamine replacement in adult rats exposed with this toxicant. In addition, Vit.E is able to compensate the adverse effects of sodium arsenite on sperm number, motility, viability and normal sperm morphological anomalies.
